# Beyond first clutches: Second broods reshape selection on breeding timing in forest and urban great tits

**DOI:** 10.1111/1365-2656.70284

**Published:** 2026-05-26

**Authors:** Jérémy Defrance, Marcel Lambrechts, Amélie Fargevieille, Samuel Perret, Arnaud Grégoire, Anne Charmantier

**Affiliations:** ^1^ CEFE, Université Montpellier, CNRS, EPHE, IRD Montpellier France

**Keywords:** great tit, laying date, multiple brooding, reproductive selection, urbanisation

## Abstract

1. Urbanisation provides a unique framework for studying how organisms respond to human‐modified environments. Although phenotypic differences between rural and urban populations are increasingly well documented, our knowledge of how natural selection operates in cities remains limited. In particular, few studies have quantified the direction and shape of natural selection acting on breeding phenology in urban environments.
2. Using a 13‐year dataset on great tits (*Parus major*), we investigated how first clutch laying date (FCLD) influences both fledgling output from first broods and total seasonal productivity (first and second clutches combined).
3. We first tested whether FCLD differed between habitats and among urban plots. We then estimated natural selection on FCLD relating breeding phenology to fledgling number from (i) first broods only and (ii) total annual productivity including first and second broods. Finally, we evaluated how FCLD and habitat jointly affected the probability of multiple brooding.
4. As in previous studies, FCLD occurred earlier in more urbanised areas. With first broods only, earlier breeding tended to be associated with higher fledging numbers in forest but not in urban habitats. In contrast, when including second clutches, a higher annual productivity was found for earlier breeders in both habitats. When controlling for clutch size, no direct selection on FCLD was detected. Earlier breeders were also more likely to initiate a second clutch, particularly in urban populations. Finally, structural equation models further showed that the effect of FCLD on annual fledgling production was largely indirect, mediated by the probability of multiple brooding and the resulting increase in annual egg number. Together, our analyses suggest that earlier laying females achieve higher annual fledgling productivity, mainly determined by a higher probability of double brooders and the resulting higher seasonal egg production in both forest and city.
5. In general, these results highlight the importance of evaluating selection using annual reproductive output rather than single breeding attempts. We believe that the underlying proximate mechanism that may promote this advantage may be related to the dynamics of prey abundance throughout the birds' breeding season, and we encourage further studies that would compare these prey dynamics across habitats.

## Graphical abstract

Most studies of selection on breeding timing consider only first clutches. Using a 13‐year dataset of urban and forest great tits, we show that including second broods reshapes estimates of natural selection, revealing that early breeding is favoured through increased probability of multiple brooding and higher annual reproductive output.

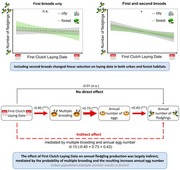

## 1 INTRODUCTION

Urban landscapes are characterised by increased loss and fragmentation of natural habitat. These landscapes are defined as areas with most of their surface covered by man‐made infrastructures (Pickett et al., 2011). Urbanisation alters key ecological conditions—including temperature, light and food availability, which can induce both short‐term plastic phenotypic responses and over longer time scales, evolutionary change (Foley et al., 2005). Urbanisation has been shown to have a significant impact on organisms on an ecological and evolutionary time‐scale (Rivkin et al., 2019; Theodorou, 2022). Therefore, it provides an interesting setting for studying how and why phenotypic traits respond to environmental changes of anthropogenic origin, which explains the increased interest of the scientific community in urban ecology and evolution (Szulkin et al., 2020). It is now widely acknowledged that natural and urban populations of the same species differ in many phenotype‐associated characteristics, such as morphology (Ofori et al., 2025), behaviour (Trigos‐Peral et al., 2024), physiology (Franco‐Belussi et al., 2024), or life history (Weinberg et al., 2025). However, despite increasing interest in urban evolutionary ecology, very few studies have explicitly quantified natural selection on these differentiated traits (Charmantier et al., 2024).

Associations between reproductive traits and the level of urbanisation have been studied in detail in a range of wild bird species (Capilla‐Lasheras et al., 2022; Sepp et al., 2018), often showing lower fledgling production in more urbanised areas compared to semi‐natural or natural habitats (Chamberlain et al., 2009; Sepp et al., 2018). Comparisons within species show that urban birds tend to lay earlier in the season and produce smaller clutches compared to birds breeding in less artificialised habitats (Chamberlain et al., 2009). Birds rely on a combination of abiotic environmental cues (e.g. perceived photoperiod, air temperature) and biotic cues such as food resources to time their breeding phenology (Gil & Brumm, 2013; Seress et al., 2018). These cues are altered in urban landscapes and therefore can influence reproductive decision‐making processes. Higher air temperatures due to the urban heat island effect and an increased exposure to artificial light (Dominoni et al., 2014; Sumasgutner et al., 2023) can advance the onset of egg laying in more urbanised environments (Dominoni et al., 2020). Additionally, the quality and quantity of food resources have been shown to differ between rural and urban landscapes (Seress et al., 2018; Sinkovics et al., 2023) and might directly or indirectly influence egg‐laying decisions via a change in parental body condition or a change in the quality or availability of food required to form eggs or rear offspring (Boutin, 1990; Robb et al., 2008). For instance, lepidopteran larvae (caterpillars), a high‐quality food source for nestling growth in many passerine species (Isaksson et al., 2008; Perrins, 1991), are only available for a short period in seasonal environments with a uniform vegetation cover (Mägi et al., 2009; Nager & van Noordwijk, 1995; Van Balen, 1973). Birds that time their nesting to match the peak abundance of caterpillars achieve higher fledgling production, while those rearing nestlings after the peak face reduced success (Burgess et al., 2018; Visser & Gienapp, 2019). Patterns of selection favouring earlier laying dates are commonly observed in natural populations of insectivorous passerines in temperate areas in the context of earlier springs because of global climate warming (Van Noordwijk et al., 1995; Visser & Gienapp, 2019). Because laying date and clutch size are considered key drivers of intraspecific variation in number of fledglings, increased urbanisation is likely to lead to new selection pressures (Donihue & Lambert, 2015; Lowry et al., 2013). However, in urban bird populations, the direction and magnitude of natural selection and the underlying mechanisms driving selection remain rarely studied and poorly understood (Badyaev et al., 2008; Charmantier et al., 2024; Corsini et al., 2021; Senar et al., 2014).

Outside urban environments, natural selection on reproductive timing has been estimated across many populations of passerines in temperate areas, in particular in blue tits *Cyanistes caeruleus* and great tits *Parus major* (De Villemereuil et al., 2020), two model species belonging to the best‐studied wildlife species in the world (Clutton‐Brock & Sheldon, 2010). Broader evidence of negative fitness consequences associated with phenological mismatches has also been documented across other taxa (Shipley et al., 2020). Although many studies have documented strong directional selection for earlier laying dates in deciduous habitats (De Villemereuil et al., 2020; Gienapp et al., 2006; Mägi et al., 2009; Van Noordwijk et al., 1995), evidence suggests that this pattern is attenuated or absent in evergreen habitats where the seasonal peak in caterpillar availability is less pronounced or more prolonged (Burgess et al., 2018; Grosbois et al., 2006). In urban settings, three studies estimated the shape and strength of reproductive selection in great tits generally reporting negative linear selection (i.e. earlier breeders producing more fledglings); although with some variation across habitats and study sites (Branston et al., 2021; Caizergues et al., 2018; Hõrak et al., 1997). Branston et al. (2021) concluded that both forest and urban populations experienced negative linear selection, consistently favouring earlier breeders. Similarly, Hõrak et al. (1997) found negative selection, although their study was conducted exclusively in an urban environment. In contrast, Caizergues et al. (2018) found no evidence for selection on breeding phenology in the city but evidence for negative selection in the forest. Furthermore, this latter study in the south of France suggested that earlier laying dates in urban environments could be maladaptive, with trait shifts in the city misaligned with selective pressures—perhaps due to the presence of a mismatch between urban artificial cues (e.g. increased temperature and altered photoperiod) and resource abundance (Caizergues et al., 2018, 2022). Taken together, these studies underscore the uncertainties surrounding the relationship between number of fledglings and laying date in urban environments, as no clear or consistent pattern has yet emerged.

Laying date may also play a role at a larger scale than a given breeding event, influencing an individual's seasonal productivity by affecting its success in all breeding attempts within a season (Bukor et al., 2022; Husby et al., 2009). Considering this perspective, a possibly important limitation of all attempts described previously to explore natural selection on avian breeding phenology in an urban context is that their fitness estimation disregarded second broods: they used the number of fledglings from first broods only. Because great tits are facultative double brooders (Bukor et al., 2022; Husby et al., 2009), we posit here that considering second broods in the fitness estimate used for selection analyses may shed a very different light on the adaptive value of reproductive strategies which are specific to rural or urban environments (Charmantier et al., 2017). Although the occurrence of second broods lacks systematic report in studies on the timing of breeding and/or selection acting upon it, it can vary greatly across a species distribution, for example from 8% to more than 60% in great tits (Husby et al., 2009; Yuta & Koizumi, 2012). Interpretations of previous findings may change drastically when accounting for the full‐season fledgling production, especially in populations with high multiple brooding prevalence. This potential bias may also mask differences across habitats if the occurrence of later clutches varies between forest and city, which is in itself a rarely explored question. Note that a study by Bukor et al. (2022) in Hungarian great tits revealed that second broods occur significantly more frequently in urban (44%) compared to forest areas (36%), while a Polish study previously showed the reverse (Luniak, 1992). Investigating how second broods contribute to the annual number of fledglings could therefore provide crucial insights into the selective pressures shaping breeding strategies in urban and forest environments. Experimental work has further highlighted the challenge of disentangling the causal effects of laying date from parental quality on reproductive success (Verhulst & Nilsson, 2008), supporting the need to consider both direct and indirect pathways by which early breeding may enhance seasonal fitness.

The present study investigates the breeding consequences of the first clutch laying date (hereafter called FCLD) in urban and rural great tits and along an urbanisation gradient in southern France. First, we hypothesise that the presence of artificial light, increased ambient temperature and/or human‐provided food should lead females to lay eggs earlier in more urbanised areas. Second, we estimate and compare reproductive selection across habitats by focusing on the number of fledglings from the first breeding attempt only as classically done. Based on previous findings (Caizergues et al., 2018), we predict a negative linear selection favouring earlier breeders in the forest habitat only. This expectation stems from habitat differences in vegetation composition and food phenology: forests dominated by deciduous oaks show a short and synchronised peak of caterpillar abundance (Perrins, 1991; Van Balen, 1973), increasing the importance of matching breeding timing with food availability (Visser & Gienapp, 2019), whereas urban habitats present more heterogeneous and extended resource phenologies, potentially weakening directional selection on laying date (Seress et al., 2018, 2020). Third, we estimate and compare reproductive selection across habitats considering the number of fledglings of both first and second clutches. Because forest and urban habitats in southern France differ in the dominant tree species (Charmantier et al., 2017; Zandt et al., 1990) and tree species differ in the peak date of caterpillars (Van Balen, 1973; Zandt et al., 1990), we expect stronger negative selection on FCLD, favouring earlier breeding, in the forest study plot dominated by broad‐leaved deciduous oak than in the city study plots mostly dominated by pine trees and evergreen oak trees favouring later clutches (Lambrechts et al., 2008; Van Balen, 1973; Zandt et al., 1990). Finally, we investigate how FCLD and habitat jointly influence the probability of initiating a second clutch and whether the effect of FCLD on annual fledgling number is mediated by multiple brooding and annual egg number, in order to better understand how breeding timing may affect reproductive output across environments.

## 2 MATERIALS AND METHODS

### 2.1 Study plots and urbanisation gradient

The breeding phenology of Great tit was monitored from 2011 to 2023 in forest and urban plots equipped with nest‐boxes suitable for great tits (Demeyrier et al., 2016). An urbanisation index was attributed to each urban nest‐box based on the proportion of impervious surface area (ISA; Thompson et al., 2025). Calculations were performed using the Imperviousness Density 2018 raster dataset (10 m resolution) from the Copernicus Land Monitoring Service (European Environment Agency, 2018; https://land.copernicus.eu/en/products/high‐resolution‐layer‐imperviousness/imperviousness‐density‐2018). The proportion of ISA was quantified in QGIS (v3.22.0) within a 100‐m diameter circular area around each nest‐box as great tits tend to occupy reduced territory size during the breeding season (Wilkin et al., 2006).

The rural study plot (La Rouvière forest, Montarnaud, 43°39′51.3″ N, 3°40′01.6″ E), mainly visited by runners and hunters, is a woodland dominated by downy oaks (*Quercus pubescens*) and evergreen oaks (*Quercus ilex*).

The urban setting consists of eight study plots across the city of Montpellier (43°36′39.7″ N, 3°52′35.3″ E), ranked here in increasing order of mean urbanisation index: the Parc du Lunaret (ZOO), Grammont (GRAM), the botanical garden (BOT), the campus of CNRS (CEF), Mosson (MOS), Font Colombe (FONT) the faculty of sciences (FAC) and Mas Nouguier (MAS; Gervais et al., 2025). Vegetation cover is highly heterogeneous across urban areas, from local tree species (e.g. evergreen oaks) to ornamental species like *Platanus*. Nest‐boxes were attached to trees in parks or along roads and therefore exposed to various levels of artificial light at night, human disturbance and pollution (Caizergues et al., 2021).

### 2.2 Great tit monitoring and data collection

Nest‐boxes were monitored at least once a week during the breeding period from mid‐March to mid‐July to record information about great tit nesting phenology: FCLD, clutch size, number of hatchlings and number of fledglings. When at least one egg was observed following a weekly control with no eggs, FCLD was back calculated assuming that the female laid one egg per day (Perrins, 1970). In our analyses, FCLD was expressed in ordinal date, where 1 = 1 January. Between 10 and 15 days after hatching, the parents were captured inside nest‐boxes in order to ring them with a uniquely numbered metal ring (CRBPO Museum Paris). Additionally, parental sex was determined on the basis of plumage characteristics and the presence of a brood patch in females. At 14 or 15 days after hatching, the nestlings were individually ringed and measured for morphological traits (i.e. tarsus length, wing length and body mass). We monitored first clutches, replacement clutches and second clutches, and the fieldwork monitoring ended every year mid‐ July after the last fledging event. First clutches (total monitored in forest: *n* = 476, urban: *n* = 1296; Table S1) were defined as breeding attempts that were referenced within an interval of 30 days following the date of the first egg observed in a given year and study area (Nager & van Noordwijk, 1995). Clutches initiated after this period were considered late clutches. These included either (i) a second breeding attempt (hereafter referred to as *second clutches*) by a female that had already been observed laying in the study area earlier in the season (successful: forest *n* = 94, urban *n* = 107; unsuccessful: forest *n* = 9, urban *n* = 26; Table S1), or (ii) a breeding attempt by a female that was not captured during the first or second reproduction in the same year (hereafter referred to as *unidentified late clutches*; forest *n* = 132, urban *n* = 368; Table S1).

### 2.3 Statistical analysis

All statistical analyses were performed with R software (version 4.2.2; R Core Team, 2016). We used linear (lm), linear mixed (lmer) and generalised linear mixed (glmer) models implemented via the lme4 (v1.1–35.1; Bates et al., 2015) and lmerTest (v3.1–3; Kuznetsova et al., 2017) packages. Type III ANOVAs were used to quantify FCLD in each habitat and to test the effects of FCLD, habitat and their interaction on the probability of initiating a second brood, via the car package (v3.1–2; Fox & Weisberg, 2011). We evaluated the model assumptions using the simulateResiduals function of the DHARMa package (v0.4.6; Hartig, 2016) and assessed multicollinearity using the vif function from the car package.

#### 2.3.1 Comparing FCLD between forest and urban habitats and along the urbanisation gradient

First, the differences between forest and urban populations in their breeding phenology were explored using a linear mixed model run on repeated FCLD from 1679 breeding events (compared to 1772 first clutches monitored, because of some missing FCLD data; Table S1), 986 with known females and 693 with unknown females. The model included as fixed effects habitat (forest or urban), year (discrete predictor with 13 levels) and the interaction between habitat and year. Second, focusing exclusively on urban breeding events (*n* = 1184, 637 with known females and 547 with unknown females; Table S1), we replaced the habitat fixed effect by the urbanisation gradient index (ISA continuous variable; see description above). This sample size is lower than the total number of first urban clutches monitored (*n* = 1296) due to missing FCLD data and missing GPS coordinates for a few older nest‐boxes (2011–2013), which prevented the calculation of the ISA values. Note that breeding events with unknown females are numerous because of frequent brood failures (due to e.g. predation or extreme climatic events) happening before parent capture. In both models, female and nest‐box identities were added as random effects to account for non‐independence of data collected on the same individuals or in the same nest‐box. In order to avoid missing fraction bias by removing all breeding records with unknown female identities, a random unique ring number was assigned to unidentified females (i.e. one different per female and breeding attempt). Note that including or removing these observations did not change the main results in the models described below.

#### 2.3.2 Estimating reproductive selection differentials and gradients and comparing them between habitats

Here, we aimed to understand how reproductive selection acts on the date of the first egg laid in a given season (i.e. FCLD). Because laying date is expressed (and varies) on a yearly basis and selection is a within‐generation process (Lande & Arnold, 1983), we chose the number of fledglings per brood as a fitness measure rather than life‐time breeding performance or number of recruits (that are mixing mother and offspring fitness; Bouwhuis et al., 2015).

For all models described in this section, the number of fledglings was standardised by dividing each observation by the overall average value within each habitat, thereby accounting for habitat‐level differences in average reproductive output while maintaining inter‐annual variation in relative fitness. To facilitate comparison of selection estimates between studies, FCLD and clutch size were also standardised using the following Equation (1).

(1) $$x_{i}=\frac{a_{i}-\bar{x}\left(a\right)}{S\left(a\right)},$$

where $x_{i}$ represents the scaled value (mean of 0 and standard deviation of 1) from the corresponding individual value $a_{i}$ for a given original trait *a*, calculated by subtracting the mean and dividing by the standard deviation of the trait within a specific population.

To estimate the force and direction of selection on FCLD in each habitat, two types of models were run. First, we calculated the linear selection differential for FCLD in each habitat separately. Selection differentials measure the total selection that combines both direct (selection acting only on the focal trait) and indirect (selection acting on traits correlated to the focal trait) selection (Lande & Arnold, 1983). The linear selection differential for FCLD $\beta_{1}$ was estimated using the following Equation (2).

(2) $$\omega =\alpha +\beta_{1}.x_{1}+\varepsilon$$

where ω represents the standardised number of fledglings (i.e. the measure of relative fitness), α the intercept, $x_{1}$ the standardised FCLD and ε the error. A negative $\beta_{1}$ implies that earlier breeding events are associated with a higher number of fledglings.

Second, we calculated the linear selection gradients for FCLD in each habitat. Selection gradients measure the direct selection on each trait taking into account indirect selection on other traits (Lande & Arnold, 1983). Such a model includes traits that are measured and potentially correlated with the focal trait. Thus, clutch size was included in these models as a negative relationship between FCLD and clutch size has been shown in this species, including in the focal population (i.e. later clutches are smaller; Charmantier et al., 2017). Here, the linear selection gradients for FCLD β_1_ and clutch size β_2_ were estimated using the following Equation (3).

(3) $$\omega =\alpha +\beta_{1}.x_{1}+\beta_{2}.x_{2}+\varepsilon$$

where ω represents the relative fitness, α the intercept, $x_{1}$ and $x_{2}$ the standardised trait value (FCLD and clutch size) and ε the error.

In addition to linear estimates, we also calculated quadratic selection differentials and gradients to evaluate non‐linear (i.e. stabilising or disruptive) selection acting on FCLD and clutch size. The quadratic selection differentials (*γ*) were estimated by adding squared standardised trait values to Equation (2), resulting in the following Equation (4).

(4) $$\omega =\alpha +\beta_{1}.x_{1}+\frac{1}{2}.\gamma_{1}.x_{1}^{2}+\varepsilon$$

where *ω* represents the relative fitness, $x_{1}$ is the standardised value of FCLD, and $\gamma_{1}$ represents the quadratic selection differential.

Quadratic selection gradients were obtained by including both the squared FCLD and the squared clutch size in the multivariate model (based on Equation 2), as follows in Equation (5).

(5) $$\omega =\alpha +\beta_{1}.x_{1}+\beta_{2}.x_{2}+\frac{1}{2}.\gamma_{1}.x_{1}^{2}+\frac{1}{2}.\gamma_{2}.x_{2}^{2}+\varepsilon$$

where $x_{1}$ and $x_{2}$ are the standardised trait value (FCLD and clutch size), $\gamma_{1}$ and $\gamma_{2}$ represent the quadratic selection gradients for each trait.

For both selection differentials and selection gradients, models were run with female identity, nest‐box number and year as random effects to control for repeated measurements within the same year and for the same individual.

Furthermore, because selection is expected to vary from year to year depending on ecological conditions, we assessed the annual variation in selection differentials and gradients on FCLD for analyses considering first‐brood fledgling production only, in both forest and urban populations. To this end, we ran linear models that included the year as a fixed effect, along with its interaction with FCLD, allowing both the intercept and the slope of the relationship between FCLD and fitness to vary over years. This approach enabled us to estimate and compare the strength and direction of selection across breeding seasons, and to evaluate how consistent selection on FCLD is over time in the forest habitat. Third and finally, in order to statistically compare selection operating in the city and the forest, the two datasets were pooled. We tested for significant differences in selection differentials and gradients (both linear and quadratic) between habitats by including the categorical variable ‘habitat’ and the interaction term *‘*habitat × FCLD’ (and *‘*habitat × FCLD^2^’ for quadratic models) in the respective equations. The significance of these interaction terms was assessed using the *t*‐test for model coefficients. All linear mixed models (linear and quadratic) otherwise had the same structure as defined earlier, with female identity, nest‐box number and year as random effects.

The models described above were run using as focal trait ($x_{1}$) the date of the first egg laid during a breeding season, and as fitness estimation (ω), the number of fledglings either from first clutches only, or from the first and second clutches pooled. In analyses that included both first and second clutches, the latter were restricted to cases where the same female was identified on two successive breeding attempts within the same season. This included females whose first attempt was either successful (forest: *n* = 86, urban: *n* = 59) or not (forest: *n* = 9, urban: *n* = 22). Unidentified late clutches, including those for which the female was not captured during the second breeding attempt or at any previous reproduction in the same year, were excluded from these analyses, as their reproductive history could not be established. For females that laid two clutches in a year, we combined the total number of eggs laid and fledglings produced from both attempts, while FCLD was always the observation of the first egg during the first attempt.

Across 13 years (2011–2023), a total of 2508 breeding attempts (combining first and later broods; see Table S1) were monitored. We removed 319 records for broods that underwent experiments that could influence either FCLD, the clutch size, or the number of fledglings (e.g. delaying FCLD, brood‐size manipulations). Second, due to the lack of female identification in later clutches (mainly unidentified late clutches), 468 records of later clutches were excluded from the analyses on both first and second clutches. Finally, we excluded 74 events due to missing information on either FCLD (mainly in 2020 during covid19 lockdown) or number of fledglings. Overall, analyses focusing exclusively on first breeding attempts included 455 forest and 1016 urban first clutches (1471 events in total; Table S1). For analyses considering annual reproductive output across first and second clutches, we additionally included 95 forest and 81 urban later breeding attempts for which female identity could be reliably assigned, resulting in a total of 1647 breeding events. Due to missing clutch‐size information, an additional 14 breeding attempts were excluded from selection gradient analyses. For first breeding attempts, the females were captured and identified in 328 forest broods (72.1%) and 558 urban broods (54.9%). In order to avoid a missing fraction bias by removing the 39.8% of breeding records with unknown female identities, a random unique ring number was assigned to unidentified females (i.e. one different per unidentified females and per breeding attempt). Note that including or removing these observations did not change the main results in the models described below.

#### 2.3.3 Assessing the influence of FCLD on the probability of initiating a second clutch and reproductive output

In order to better understand the relation between breeding phenology and multiple brooding in both habitats, we assessed the probability of initiating a second clutch using a generalised linear mixed model with a binomial response variable. Habitat, FCLD and their interaction were included as fixed effects. Female identity, nest‐box number and year were also added as random effects to account for repeated measures and nonindependence of data across individuals and years.

Finally, to assess whether selection on FCLD is mediated by multiple brooding, we conducted two complementary analyses: (1) a structural equation model (SEM) to estimate direct and indirect effects of FCLD on seasonal reproductive output via multiple brooding and annual egg number, separately for each habitat and (2) linear selection gradient models including *multiple brooding* as a covariate, again separately for each habitat. Methodological details for this second analysis are provided in Supporting Information: Appendix 1. For SEM, we used a piecewise approach, implemented in R with the package piecewiseSEM (v2.1.2; Lefcheck, 2016). This framework allows for the integration of multiple linear (or generalised linear) mixed models into a single causal network while accounting for nonindependence and hierarchical data structure. We defined the hypothesised causal structure based on biological expectations and previous findings:

1. Multiple brooding (binary response: 0 = single brood, 1 = multiple broods) was modelled as a function of FCLD, using a binomial generalised linear mixed model (GLMM).
2. Annual egg number (standardised) was modelled as a function of FCLD and multiple brooding, using a linear mixed model (LMM).
3. The annual number of fledglings (standardised, total throughout the season) was modelled as a function of FCLD and the annual egg number, using an LMM.

All models included female identity, nest‐box number and year as random intercepts. These models were fitted separately for forest and urban habitats using the sem() function. Each submodel captures a different component of the overall causal structure: model 1 estimates the direct effect of FCLD on the probability of multiple brooding; model 2 estimates the direct effects of FCLD and multiple brooding on annual egg number; and model 3 estimates the direct effects of FCLD and annual egg number on seasonal fledgling number. Together, these models allow us to quantify the direct effect and indirect effects of FCLD on reproductive output: (i) via annual egg number alone and (ii) via multiple brooding followed by annual egg number (Figure S1).

### 2.4 Ethical considerations

All procedures complied with current French legislation. Birds were captured and ringed under permits delivered by the Centre de Recherches sur la Biologie des Populations d'Oiseaux (CRBPO, Muséum National d'Histoire Naturelle, Paris). This study did not require approval from an animal ethics committee.

## 3 RESULTS

### 3.1 Breeding trait differences between forest and urban habitats

**FIGURE 1:**
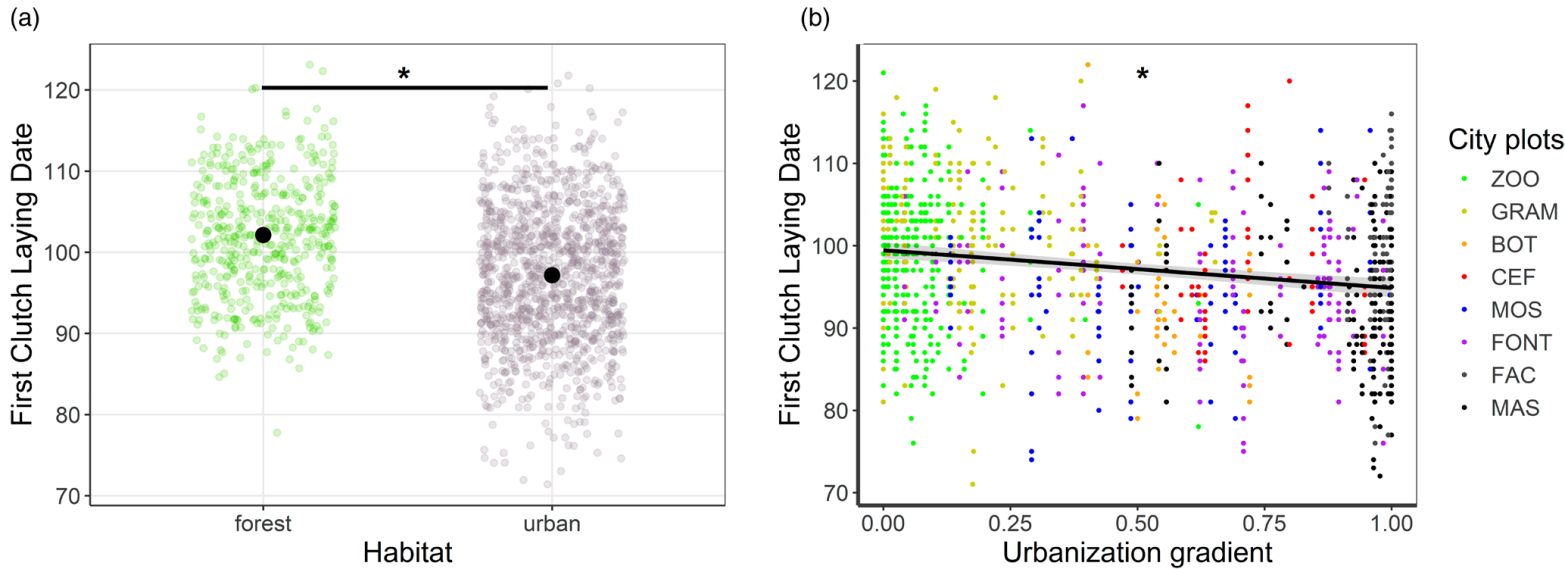
Differences in great tit laying phenology between forest and city habitats (a) and along the urbanisation gradient within the city (b). The urbanisation index varied from 0 (less urbanised areas) to 1 (most urbanised areas). *p < 0.05.

Our results confirm previous findings that urban females lay significantly earlier than their forest conspecifics (on average 4.50 days earlier, *F*_1_ = 6.551, *p* < 0.05, Table S2; Figure 1a).

Also, as expected, when focusing on the within‐city level, a negative correlation was found between FCLD and the urbanisation index (*F*_1_ = 4.577, *p* < 0.05, Table S3; Figure 1b). On average, females breeding in most urbanised territories laid 4.54 days earlier than females in the least urbanised areas of Montpellier.

In both analyses, forest and urban great tits showed strong annual fluctuations in their FCLD (model with habitat: *F*_12_ = 26.641, *p* < 0.001, Table S2; model with urbanisation index: *F*_12_ = 14.584, *p* < 0.001, Table S3). Furthermore, the divergences of FCLD between habitats also varied annually (habitat × year, *F*_12_ = 4.337, *p* < 0.001, Table S2; Figure S2; urbanisation index × year, *F*_12_ = 2.119, *p* < 0.05, Table S3).

### 3.2 Comparison of reproductive selection between habitats using first‐brood fitness

**FIGURE 2:**
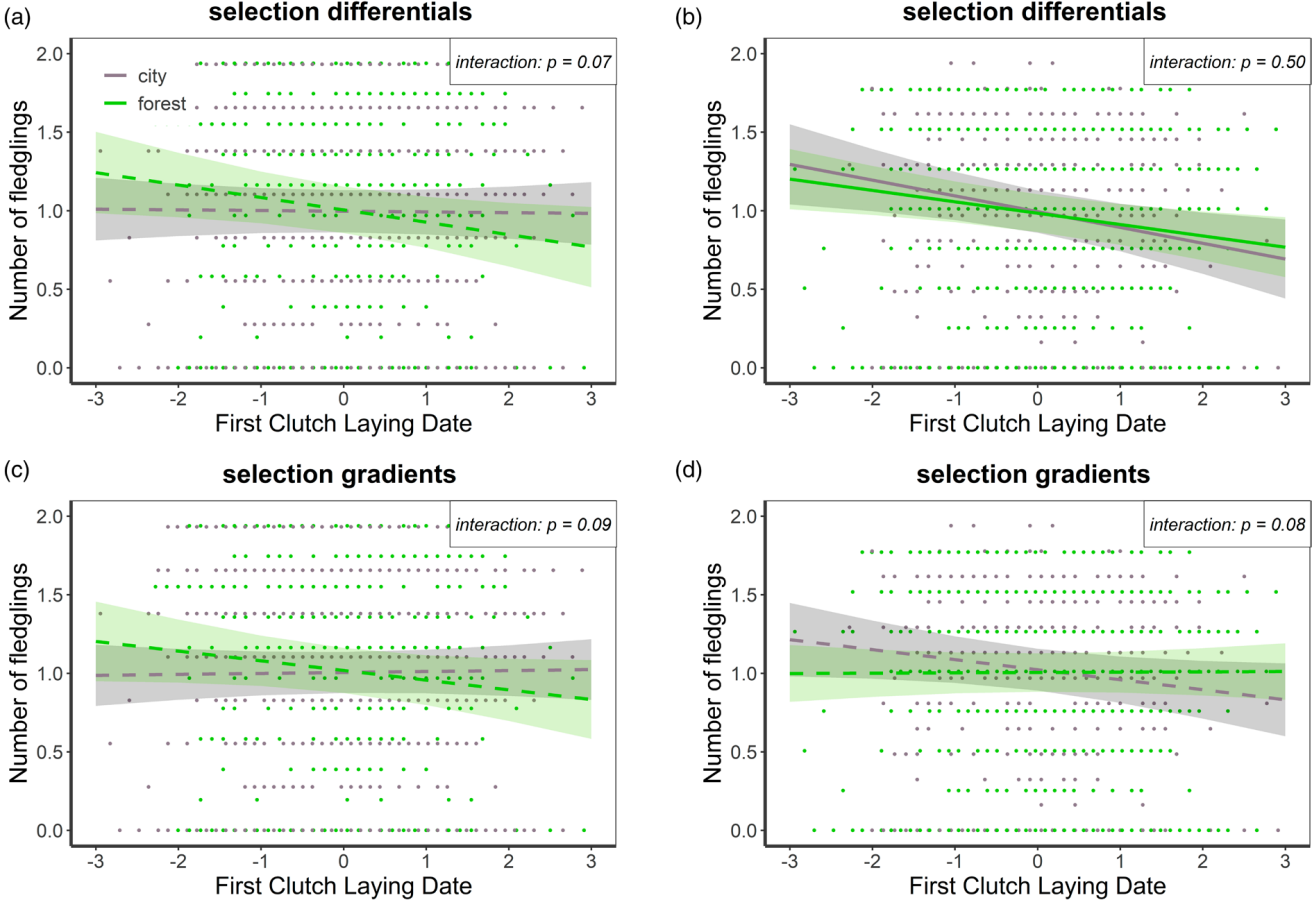
Linear selection differentials (a, b) and gradients (c, d) for standardised first clutch laying date (FCLD) in forest and urban habitats, following the analysis derived from the full model. Panels a and c represent estimates for first clutches only, while panels (b) and (d) include both first and second clutches. Solid lines indicate significant effects, and dashed lines indicate non‐significant effects. Shaded ribbons represent 95% confidence intervals.

Following the analysis on the first clutches alone, there was a tendency for a negative linear selection differential in the forest, while there was no evidence of any linear selection in the city (habitat‐ specific models, forest: $\beta_{1}$ = −0.066 ± 0.039, *p* = 0.09; urban: $\beta_{1}$ = −0.011 ± 0.026, *p* = 0.67; Figure 2a; Table 1a). The interaction between FCLD and habitat in the full model was marginally significant (estimate = −0.075 ± 0.041, *p* = 0.067; Table 1a), suggesting that the selection tended to be stronger in the forest. Including both FCLD and clutch size in the selection gradients model showed a negative, yet non‐significant, linear selection gradient in the forest and no selection in the city (habitat‐specific models, forest: $\beta_{1}$ = −0.046 ± 0.038, *p* = 0.23; urban: $\beta_{1}$ = −0.001 ± 0.025, *p* = 0.96; Figure 2c; Table S4a). There was a marginal interaction between FCLD and habitat (estimate = −0.068 ± 0.040, *p* = 0.09; Table S4a).

**TABLE 1:** Linear selection differentials for the standardised first clutch laying date (FCLD), based on an estimate of reproductive output that includes (a) first clutches only and (b) first and second clutches together.

Fitness estimation	Habitat	Sample size	Linear selection differentials					Difference between habitats				
			Estimate	Std. error	d.f.	*t* value	pr (>|*t*|)	Estimate	Std. error	d.f.	*t* value	pr (>|*t*|)
(a) First clutches	Forest	455	*−* *0.066*	*0.039*	*325.402*	*−1.718*	*0.087*	*−0.075*	*0.041*	*1395.000*	*−1.832*	*0.067*
	Urban	1016	−0.011	0.026	961.105	−0.428	0.669	—	—	—	—	—
(b) All clutches	Forest	455	**−0.120**	**0.041**	**300.308**	**−2.953**	**0.003**	0.028	0.042	1423.000	0.679	0.497
	Urban	1016	**−0.069**	**0.026**	**927.535**	**−2.643**	**0.008**	—	—	—	—	—

*Note*: Linear selection differentials were estimated using models fitted separately for each habitat (left‐hand side of table), while the differences between habitats were tested using a model including both habitats (right‐hand side of table). Significant effects (*p* < 0.05) and trend (0.05 < *p* < 0.10) effects are shown in bold and italics and were tested using linear mixed models.

To assess temporal consistency in selection on FCLD, we estimated year‐specific selection differentials and gradients based on first broods only in both forest and urban populations. Although the strength of the selection varied among years in both habitats, significant FCLD × year interactions were detected in the forest population but not in the urban population, indicating greater inter‐annual variability in the selection on the laying date in the forest than in the city (Table S8; Table S9).

### 3.3 Comparison of reproductive selection between habitats combining first and second clutches

When using first and second clutches combined to estimate fitness, there was a significant negative linear selection differential in both habitats favouring earlier breeders (habitat‐specific models, forest: $\beta_{1}$ = −0.120 ± 0.041, *p* < 0.01; urban: $\beta_{1}$ = −0.069 ± 0.026, *p* < 0.01; Figure 2b; Table 1b). The interaction between FCLD and habitat, tested using the full model (combining datasets from both habitats and including habitat as a variable and the interaction term FCLD × habitat), was not significant (estimate = 0.028 ± 0.042, *p* = 0.50; Table 1b). When including both FCLD and clutch size in habitat‐specific selection gradient models and considering both first and second clutches combined, there was no evidence of directional selection on FCLD in either the forest or the city, although estimated gradients were negative in both habitats (forest: $\beta_{1}$ = −0.048 ± 0.034, *p* = 0.15; urban: $\beta_{1}$ = −0.007 ± 0.024, *p* = 0.76; Figure 2d; Table S4b). The interaction between FCLD and habitat in the full model, showed a tendency towards stronger selection gradients in the forest (estimate = −0.066 ± 0.037, *p* = 0.08; Figure 2d; Table S4b).

Additionally, models estimating quadratic selection differentials and gradients revealed no evidence of significant non‐linear selection on FCLD or clutch size in either habitat (Figures S5 and S6).

### 3.4 Influence of FCLD on multiple brooding probability and reproductive output

**FIGURE 3:**
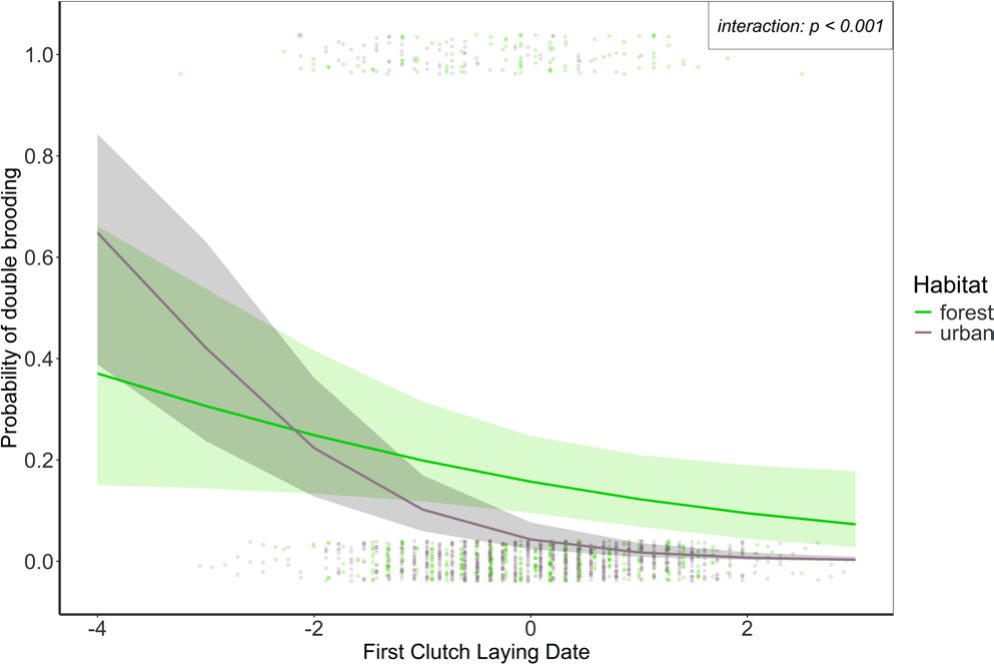
Probability of multiple brooding in urban (grey) and forest (green) great tits across their first clutch laying date (FCLD), based on the output of a generalised linear mixed model (GLMM). The model included the probability of initiating a second clutch as a binomial response variable (1 = initiated a second clutch, 0 = did not), with standardised FCLD of the first attempt, habitat (forest or urban) and their interaction as fixed effects. Female identity, nest‐box number and year were included as random effects to account for repeated measures and inter‐annual variability.

In Montpellier and surrounding study areas, 33.1% of forest and 27.9% of urban breeding events were later broods (combining true second broods and unidentified late clutches, Table S1). We found a significant interaction between FCLD and habitat on the probability of initiating a second clutch (estimate = −0.64 ± 0.18, *p* < 0.001; Figure 3; Table S7), indicating that the effect of FCLD on multiple brooding differed between habitats. In both habitats, earlier breeders were more likely to initiate a second brood, but this effect was stronger in urban areas. For instance, in the city, the probability of a second brood decreased from about 66% for the earliest clutches to nearly 0% for the latest ones, while in the forest it declined from about 39% to 5% (Figure 3).

**FIGURE 4:**
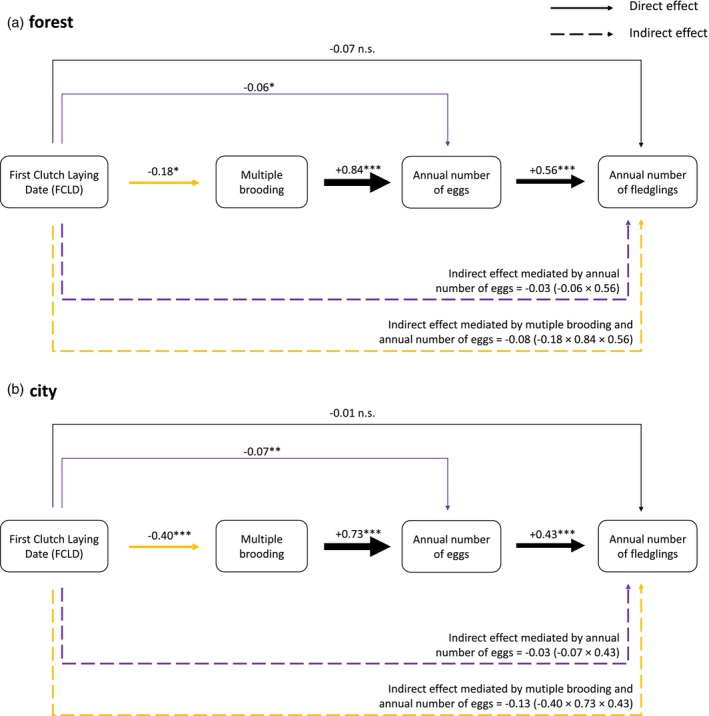
Path diagram representing the structural equation model (SEM) linking first clutch laying date (FCLD), multiple brooding, the annual number of eggs and the annual number of fledglings in forest (a) and urban (b) habitat. Solid arrows represent direct effects, while dashed arrows illustrate indirect effects mediated by other variables. Arrow colours indicate the type of indirect pathway linking FCLD to the number of fledglings: Violet arrows represent the indirect effect of FCLD via the annual egg number, and yellow arrows represent the indirect effect of FCLD via multiple brooding and the annual egg number. The arrows from FCLD to mediators are coloured to match their respective pathways. Arrow thickness is proportional to the standardised path coefficients (from Table S10), which are indicated alongside each arrow. Statistical significance is shown for direct paths using the following notation: N.s. (*p* ≥ 0.05), * (*p* < 0.05), ** (*p* < 0.01) and *** (*p* < 0.001). The diagram highlights both direct and mediated effects by which laying phenology influences reproductive output.

To further assess whether selection on laying date is mediated by multiple brooding, we conducted both a structural equation model and a selection gradient analysis that included multiple brooding as a covariate. Structural equation models showed that earlier laying increased the probability of multiple brooding (forest: std. estimate = −0.18; city: −0.40), which strongly increased clutch size (forest: +0.84; city: +0.73) and ultimately fledgling number (forest: +0.56; city: +0.43; Figure 4; Table S10). Decomposing these pathways revealed that the effect of FCLD on fledgling output was largely mediated by indirect pathways, accounting for approximately 63% of the total effect in the forest and 94% in the city (calculated as the ratio of indirect to total effects), suggesting that the direct effect of FCLD was minimal. These results are further supported by the multiple regression analysis including multiple brooding as an additional predictor, which yielded consistent selection gradients (see Supporting Information: Appendix 1; Table S11).

## 4 DISCUSSION

In the present study, we investigate the relationship between fledgling productivity and first clutch laying date (FCLD) in forest and urban great tits using long‐term data including multiple breeding attempts. We find that urban females start to lay significantly earlier than their forest counterparts and that within the city, increased urbanisation is associated with earlier FCLD. Selection analyses on first broods alone indicate habitat‐specific patterns with a tendency for a negative selection favouring earlier breeders in the forest, while there is no evidence for selection in the city. Interestingly, when accounting for the overall seasonal productivity of fledglings combining first and second clutches, selection consistently favours earlier breeding in both urban and rural study populations. Structural equation models further indicate that this relationship is largely mediated by the higher probability of initiating multiple broods among early breeders, which in turn, increases clutch size and ultimately seasonal fledgling production. We draw here lessons from this unique comparison, discussing the underlying mechanisms explaining the observed patterns and the implications on our understanding of the evolution of life‐history traits in urban environments.

Our findings of earlier FCLD in more urbanised environments are consistent with previous published results in great tits and other bird species (Chamberlain et al., 2009; Damiani et al., 2025; Sepp et al., 2018). Within the city of Montpellier, FCLD is negatively correlated with the level of urbanisation estimated by the proportion of impervious surface around each nest‐box (Table S3; Figure 1b). The advanced onset of laying in the more urbanised areas has been proximately attributed to human‐modified environmental cues, such as the length of the photoperiod altered by the presence of artificial light, ambient temperature influenced by human mineral‐based constructions (e.g. heat island effects) and the earlier availability of (artificial) food in late winter and/or early spring (Dominoni et al., 2020; Gil & Brumm, 2013; Seress et al., 2018; Sinkovics et al., 2023).

In a first set of selection analyses considering the fledging productivity of first broods only (Table 1a and Table S4a), we find no evidence for selection favouring earlier laying in the city of Montpellier, consistent with the first‐brood findings presented in Caizergues et al. (2018). Negative selection favouring earlier FCLD to maximise the number of fledglings during the first seasonal breeding attempt is a classical result found in forest bird populations, especially in broad‐leaved environments with low tree species diversity (De Villemereuil et al., 2020; Gienapp et al., 2006; Van Noordwijk et al., 1995). Interestingly, our selection differentials were weaker than those previously estimated by Caizergues et al. (2018) in the same great tit population on a shorter timescale, and also weaker than those observed in Scottish urban and forest great tits (Branston et al., 2021). By explicitly modelling year‐specific selection gradients, we find substantial inter‐annual variation in selection on FCLD, with some years showing strong negative selection and others showing weak or no directional selection (Table S8). On the contrary, the selection patterns in the urban population appear relatively consistent across years (Table S9), suggesting that the ecological conditions shaping the fitness consequences of the laying date may be more temporally stable in the city. This relative inter‐annual stability may explain why selection estimates in urban habitat remain comparable to those previously reported by Caizergues et al. (2018), while greater temporal variability in the forest could account for the slight differences observed between studies. This inter‐annual variability suggests that the fitness consequences of early breeding depend on annual environmental conditions. One explanation for this inter‐annual variability is that during years with extreme weather conditions, which are becoming increasingly frequent, the earlier first broods might have suffered from higher nestling mortality in some breeding seasons. In particular, low ambient temperatures combined with heavy rain could substantially increase nestling mortality in early broods (e.g. Zajac, 1995). This stands in contrast to findings in other systems, where late‐season extreme climatic events (such as heatwaves) have been shown to disproportionately affect late broods and thereby strengthen selection for early breeding (Marrot et al., 2017). Hence, the timing of extreme climatic events may critically shape context‐dependent selection on breeding phenology.

In a second set of selection analyses, we considered females' overall annual fledgling production (i.e. the total number of fledglings produced across all clutches in a season), with FCLD as the focal trait. Including these second clutches in our analyses significantly changed the strength of directional selection on FCLD. Note that the shift towards stronger selection favouring earlier breeding phenology is more pronounced in urban sites, despite the lower frequency of double brooding in the city. We posit that a key environmental feature to consider when interpreting both the absence of negative directional selection when considering first broods alone and relatively high fitness impact of double brooding in the urban context, is the vegetation composition and associated temporal variability in urban landscapes. It is well known that defoliating caterpillars, the optimal prey for great tit nestlings, are abundant during a short period of the year for any given host‐plant species, when plant and tree leaves are growing (Burgess et al., 2018; Nager & van Noordwijk, 1995). Therefore, the level of synchronisation between the peak date of caterpillar availability and the nestling stage in determining fledgling productivity will become more important in environments with a single dominant vegetation type, such as a forest dominated by broad‐leaved deciduous oak that favours earlier first broods (e.g. Blondel et al., 2006). However, the availability of defoliating caterpillars does not necessarily decline along the breeding season in an urban mosaic of vegetation types that combine early and late phenologies, so that the probability of a nestling mismatch with the peak date of local caterpillar availability, or alternative prey item, will become less likely (Seress et al., 2020). In our study areas of Montpellier, vegetation is predominantly composed of resinous trees (*Pinus* spp.) and evergreen oaks (*Quercus ilex*), both characterised by a later seasonal phenology (Blondel et al., 2006; Van Balen, 1973). These tree types may extend the period of food availability, potentially supporting larger second clutches in urban areas, a phenomenon observed in areas dominated by oak trees (Lambrechts et al., 2008). Beyond prey abundance and phenology, recent work has shown that the nutritional composition of insect prey (particularly lipid and protein content) varies across taxa and habitats and can strongly influence nestling development and survival (Andersson et al., 2015; Pollock et al., 2017; Twining et al., 2016, 2018). In this context, the diversity of urban vegetation can provide not only a temporally extended prey supply, but also a more heterogeneous nutritional landscape, which could buffer the cost of phenological mismatches. Consequently, urban habitats with such mixed vegetation may enable greater reproductive output for birds engaging in later broods, which could help explain the stronger shift in selection observed in the city when these later clutches are included in the analysis. Future studies integrating both temporal and nutritional dimensions of prey availability would provide a more mechanistic understanding of how urban environments shape fledgling productivity and selection on breeding phenology. Selection gradients controlling for indirect selection on FCLD via clutch size (Lande & Arnold, 1983), reveal remarkably different patterns from the selection differentials, whereby both types of analyses (first brood versus full annual reproductive output) fail to show evidence for direct selection acting on FCLD (Table S4b). The contrast between selection differentials and gradients suggests that the fitness benefits associated with earlier laying dates are driven by an increased total number of eggs laid throughout the breeding season. Since earlier laying dates are associated with a higher number of fledglings per season in analyses that include multiple broods, but not in those focused solely on first broods, it is likely that early breeders also have a higher probability of producing more than one clutch. Our findings in the final step of our analyses support this hypothesis, since they reveal that earlier breeders are more likely to initiate multiple broods compared to later breeders in both forest and urban environments, with a stronger effect of laying phenology on multiple brooding in the urban environment (Figure 3; Table S7). This correlation aligns with previous studies in various avian species (Bukor et al., 2022; Charmantier et al., 2017; Hoffmann et al., 2015; Verhulst & Nilsson, 2008) and, when considered alongside our selection analyses, suggests that initiating multiple clutches enhances annual breeding productivity (Bukor et al., 2022; Hoffmann et al., 2015). Consistent with this interpretation, both the structural equation model (SEM) and the reanalysis of selection gradients including multiple brooding (methods and results in Appendix 1; Table S11) confirmed a strong positive effect of multiple brooding on total fledgling output. In the SEM, multiple brooding had a substantial positive effect on annual egg number in both habitats and mediated a large part of the relationship between FCLD and reproductive output (Table S10). In particular, the modelling showed a very limited and insignificant direct effect of FCLD on reproductive output, as already observed in selection gradient analyses excluding multiple brooding. Biologically, this indicates that early laying does not directly enhance fledgling production within a given brood, but instead increases the likelihood of extending the reproductive period through additional broods, thereby increasing total annual reproductive output. Decomposition of the SEM pathways further revealed that these indirect effects accounted for the majority of the total effect of FCLD on fledgling production (approximately 63% in the forest and 94% in the city), confirming that the relationship between laying date and reproductive output is almost entirely mediated by correlated reproductive traits, especially in urban environments. This result also clarifies the apparent discrepancy between selection differentials and selection gradients. While selection differentials capture the total association between laying date and fitness, including both direct and indirect effects, selection gradients isolate the direct effect of laying date while controlling for correlated traits. The absence of significant selection gradients on FCLD is therefore consistent with the SEM results, which show that its effect on reproductive output is primarily indirect. Although we show here that natural selection favours the production of more than one brood per season, the prevalence of multiple brooding in our study area remains modest with 33.1% of multiple brooders in the forest habitat and 27.9% in the city (Table S1). These figures are likely conservative, as some multiple brooders may have gone undetected when subsequent attempts occurred outside monitored nest‐boxes or when nest desertion prevented parental capture. Note, however, that our recapture rates are high and very similar between the forest (0.73, 95% CI [0.63–0.79]) and the urban (0.72, 95% CI [0.64–0.79]) monitored populations (Caizergues et al., 2022).

The present study aimed to assess the force and direction of reproductive selection on the phenology of breeding initiation in urban and forest great tits, with a focus on assessing whether double brooding mattered for this purpose. Together, our findings suggest that the estimated strength of reproductive selection depends on the reproductive metric considered, with a stronger selection favouring early breeding when considering the number of fledglings per breeding season rather than from the first breeding attempt alone. We hypothesise that habitat‐specific temporal variation in the abundance and timing of key food resources, such as caterpillars, plays a crucial role in this framework. In future explorations, documenting temporal variation in resource availability would help test whether the selection on FCLD observed in urban populations results from a temporally fragmented or extended caterpillar peak across heterogeneous vegetation types. Such data would provide deeper insights into how environmental conditions shape reproductive timing and success in urban bird populations. From a biogeographic perspective, breeding seasons generally become shorter and more temporally constrained towards higher latitudes, whereas tropical and Mediterranean climates are characterised by milder conditions and potentially extended breeding opportunities (Lack, 1968; Martin, 1996). In such systems, the production of multiple broods is frequent and contributes to enhancing the annual reproductive success (Bukor et al., 2022). However, multiple brooding is not restricted to southern regions. Substantial rates of second broods have also been reported in more temperate climates, such as in Hungary (e.g. 36% of second broods in Bukor et al., 2022), sometimes even exceeding those observed in Mediterranean populations. This suggests that the occurrence of multiple brooding is not solely determined by latitude or broad climatic zones, but rather reflects local ecological conditions and environmental variability.

## AUTHOR CONTRIBUTIONS

Anne Charmantier, Marcel Lambrechts and Arnaud Grégoire conceived and funded the study. Anne Charmantier, Marcel Lambrechts, Arnaud Grégoire, Amélie Fargevieille, Samuel Perret and Jérémy Defrance collected field data. Jérémy Defrance performed the statistical analyses. Jeremy Defrance wrote the first draft of the manuscript. All the authors contributed to improving the manuscript and gave final approval for publication.

## CONFLICT OF INTEREST STATEMENT

We declare no conflict of interest.

## Supporting information


**Appendix 1.** Supplementary Methods and Results.
**Appendix 2.** Supplementary Tables and figures.
**Table S1.** Number and percentage of breeding attempts by brood type (first clutches, second clutches and unidentified late clutches with non‐captured females) in forest and urban habitats.
**Table S2.** Testing for a difference in first clutch laying date (FCLD) between forest and urban great tits.
**Table S3.** Correlation between first clutch laying date (FCLD) and the urbanisation index in the city.
**Figure S2.** Average annual first clutch laying date (FCLD) in the forest (in green) and in the city (in grey) across the monitoring years (2011 to 2023).
**Table S4.** Linear selection gradients for first clutch laying date (FCLD) based on an estimate of reproductive output that included (a) first clutches only and (b) first and second clutches together.
**Table S5.** Quadratic selection differentials for first clutch laying date (FCLD) based on an estimate of reproductive output that included (a) first clutches only and (b) first and second clutches together.
**Table S6.** Quadratic selection gradients for first clutch laying date (FCLD) based on an estimate of reproductive output that included (a) first clutches only and (b) first and second clutches together.
**Table S7.** Determinants of the probability of multiple brooding in urban and forest great tits.
**Table S8.** Linear mixed models estimating year‐specific selection differentials and selection gradients on standardised first clutch laying date (FCLD) based on annual fledgling production from first broods only in forest population.
**Table S9.** Linear mixed models estimating year‐specific selection differentials and selection gradients on standardised first clutch laying date (FCLD) based on annual fledgling production from first broods only in urban population.
**Table S10.** Standardised path coefficients from structural equation models (SEMs) conducted separately in forest and urban habitats.
**Table S11.** Linear selection gradients for first clutch laying date (FCLD), clutch size (CS) and multiple brooding (MB) based on reproductive output including all clutches.

## Data Availability

Data supporting this study are available from the IndoRes platform https://doi.org/10.48579/PRO/DYC1KO (Defrance et al., 2026a). The code used for the analyses is archived on Zenodo https://doi.org/10.5281/zenodo.19696645 (Defrance et al., 2026b).
