# Supplementary material for: Beyond first clutches: Second broods reshape selection on breeding timing in forest and urban great tits
Source: J Anim Ecol. 2026 May 26;95(7):1163–77. doi: 10.1111/1365-2656.70284 (PMC13322181; doi:10.1111/1365-2656.70284)
Supplement: Supplementary file 1 — Appendix 1. Supplementary Methods and Results. Appendix 2. Supplementary Tables and figures. Table S1. Number and percentage of breeding attempts by brood type (first clutches, second clutches and unidentified late clutches with non‐captured females) in forest and urban habitats. Table S2. Testing for a difference in first clutch laying date (FCLD) between forest and urban great tits. Table S3. Correlation between first clutch laying date (FCLD) and the urbanisation index in the city. Figure S2. Average annual first clutch laying date (FCLD) in the forest (in green) and in the city (in grey) across the monitoring years (2011 to 2023). Table S4. Linear selection gradients for first clutch laying date (FCLD) based on an estimate of reproductive output that included (a) first clutches only and (b) first and second clutches together. Table S5. Quadratic selection differentials for first clutch laying date (FCLD) based on an estimate of reproductive output that included (a) first clutches only and (b) first and second clutches together. Table S6. Quadratic selection gradients for first clutch laying date (FCLD) based on an estimate of reproductive output that included (a) first clutches only and (b) first and second clutches together. Table S7. Determinants of the probability of multiple brooding in urban and forest great tits. Table S8. Linear mixed models estimating year‐specific selection differentials and selection gradients on standardised first clutch laying date (FCLD) based on annual fledgling production from first broods only in forest population. Table S9. Linear mixed models estimating year‐specific selection differentials and selection gradients on standardised first clutch laying date (FCLD) based on annual fledgling production from first broods only in urban population. Table S10. Standardised path coefficients from structural equation models (SEMs) conducted separately in forest and urban habitats. Table S11. Linear selection gradients for first clutch lay [file JANE-95-1163-s001.docx]

**Supplementary materials**

**Appendix 1: Supplementary Methods and Results**

**Supplementary Methods** - Selection Gradients Including Multiple Brooding

To test whether selection on first-clutch laying date (FCLD) is mediated by multiple brooding, we re-ran the linear selection gradient models by including the variable multiple brooding (1 = female laid a second clutch; 0 = single clutch only) as a fixed effect, in order to disentangle fitness effects associated with breeding timing, clutch size, and the production of multiple broods. This analysis was performed separately for each habitat (Table S11).

**Supplementary results -** Selection Gradients Including Multiple Brooding

Adding multiple brooding status (0 = single clutch, 1 = second clutch) as a fixed effect in the selection gradient models revealed a strong positive effect of multiple brooding on annual fledgling output in both habitats (forest: estimate = 0.553 ± 0.121, p < 0.001; city: 0.357 ± 0.118, p = 0.003); while the direct effect of FCLD was not significant in both the forest (estimate = -0.054 ± 0.033, p = 0.10), and the city (estimate = -0.004 ± 0.024, p = 0.88; Table S9).

**Appendix 2: Supplementary Tables and figures**

**Table S1.** Number and percentage of breeding attempts by brood type (first clutches, second clutches, and unidentified late clutches with non-captured females) in forest and urban habitats. *Data are presented for the complete monitoring dataset and for the subsets used in the analyses of* between-habitat First Clutch Laying Date (FCLD)*,* within-city FCLD along the urbanisation gradient, selection differentials*, and* selection gradients*. Each row reports the number of breeding attempts and their percentage relative to the total for each habitat and dataset type. For the* selection analyses *only, the number of individual females that laid a single clutch during the season (*nbr. 1-clutch ♀*) is also reported. These counts correspond exclusively to females included in the selection datasets (tables S4, S5, S6, S8 and S9). For the selection analyses, only first clutches and later clutches with known outcomes and identified females were retained (i.e., second clutches). Unidentified late clutches, for which the female’s identity was unknown either at the time of the first or the later attempt, were excluded because their breeding history could not be reliably established. This filtering strongly reduced the number of second clutches available for analysis, especially in the urban habitat, where lower field effort during the early years of monitoring (2011–2012) limited female identification.*

|  | **monitoring sample size** | | |  |
| --- | --- | --- | --- | --- |
| **type of brood** | **forest** |  | **urban** |  |
| first clutch | 476 (67.2%) |  | 1296 (72.2%) |  |
| second clutch | 103 (14.5%) |  | 133 (7.4%) |  |
| unidentified late clutch | 132 (18.3%) |  | 368 (20.4%) |  |
| **total** | **711** |  | **1797** |  |
|  | **between-habitat FCLD analysis sample size** | | |  |
| **type of brood** | **forest** |  | **urban** |  |
| first clutch | 475 |  | 1204 |  |
|  | **along-gradient FCLD analysis sample size** | | |  |
| **type of brood** | **forest** |  | **urban** |  |
| first clutch | - |  | 1184 |  |
|  | **selection differentials analysis sample size** | | |  |
| **type of brood** | **forest** |  | **urban** |  |
|  | **nbr. attempt** | **nbr. 1-clutch ♀** | **nbr. attempt** | **nbr. 1-clutch ♀** |
| first clutch | 455 (82.7%) | 360 | 1016 (92.6%) | 935 |
| second clutch | 95 (17.3%) | - | 81 (7.4%) | - |
| unidentified late clutch | - | - | - | - |
| **total** | **550** | **-** | **1097** | - |
|  | **selection gradients analysis sample size** | | |  |
| **type of brood** | **forest** |  | **urban** |  |
|  | **nbr. attempt** | **nbr. 1-clutch ♀** | **nbr. attempt** | **nbr. 1-clutch ♀** |
| first clutch | 448 (82.5%) | 360 | 1009 (92.6%) | 935 |
| second clutch | 95 (17.5%) | - | 81 (7.4%) | - |
| unidentified late clutch | - | - | - | - |
| **total** | **543** | **-** | **1090** | - |

**
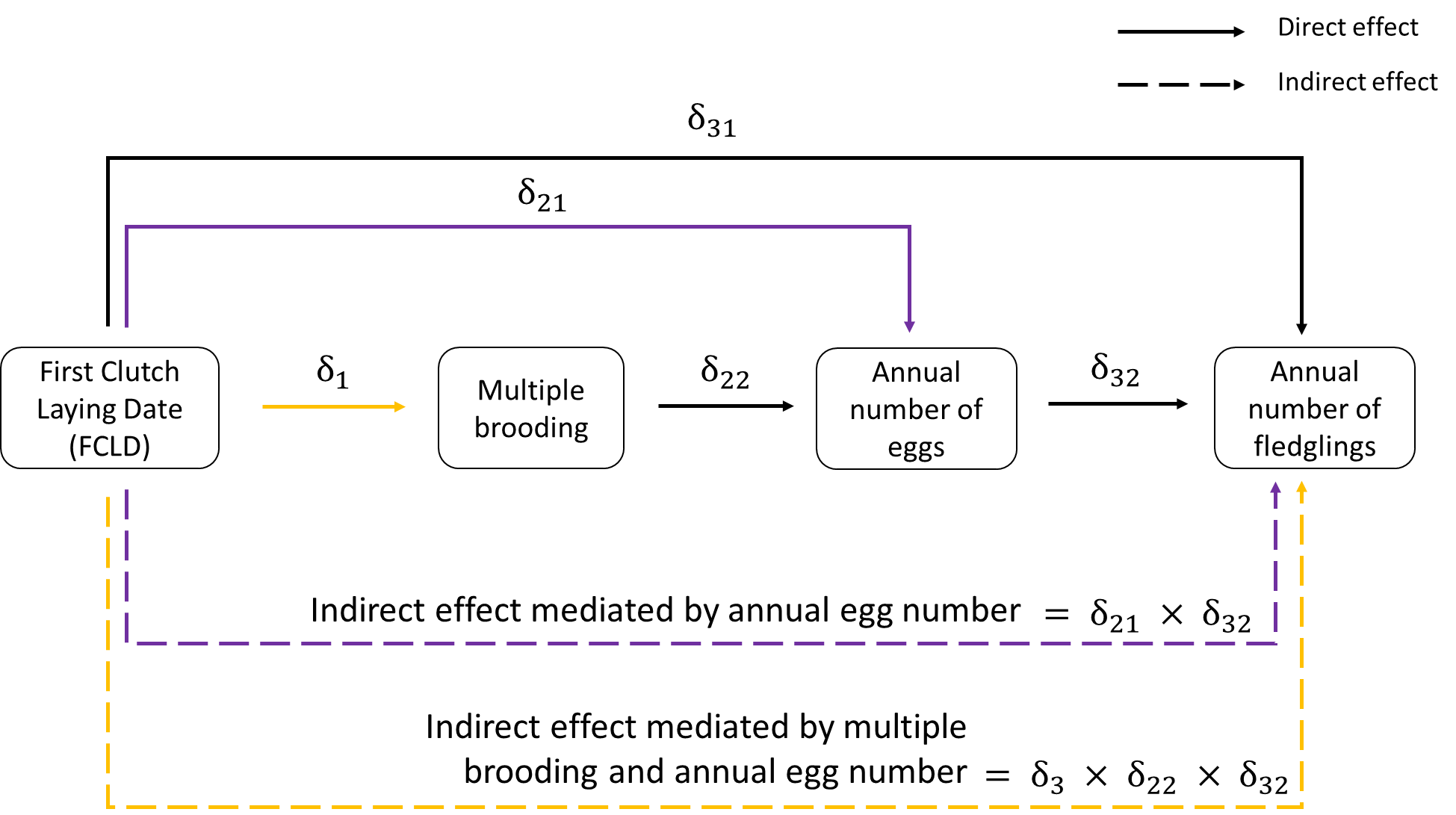
**

Figure S1. Path diagram representing the hypothesized causal structure used in the structural equation models (SEMs) to assess the direct and indirect effects of First Clutch Laying Date (FCLD) on seasonal reproductive output in forest (a) and urban (b) habitats. The model includes three linked submodels: $\delta_{1}$ corresponds to the direct effect estimated in submodel 1 (multiple brooding as a function of FCLD), $\delta_{21}$ and $\delta_{22}$ are from submodel 2 (annual egg number as a function of FCLD and multiple brooding), and $\delta_{31}$ and $\delta_{32}$ correspond to the direct effects estimated in submodel 3 (annual fledgling number as a function of FCLD, and annual egg number). All $\delta$ coefficients shown in the diagram are standardized path coefficients. Solid arrows indicate direct effects while dashed arrows represent indirect effects, which were calculated as the product of the corresponding direct effects along each path. Specifically, the indirect effect of FCLD via annual egg number corresponds to $\delta_{21}$× $\delta_{32}$ (violet path), while the indirect effect of FCLD via multiple brooding and annual egg number corresponds to $\delta_{1}$ × $\delta_{22}$ × $\delta_{32}$ (yellow path).

**Table S2**. Testing for a difference in First Clutch Laying Date (FCLD) between forest and urban great tits. *Significant effects (in bold) were tested using type III Anova performed on the results of a linear mixed model (LMM). The model included* FCLD *(expressed as Julian day, with 1 = January 1st) as the response variable, and habitat (forest or urban), year (discrete predictor with 13 levels) and their interaction as explanatory variables. Female and nest-box identity were added as random effects.*

|  | **FCLD** |  |  |
| --- | --- | --- | --- |
|  | **forest (n = 475)** | **urban (n = 1204)** | |
| mean ± s.d. | 101.63 ± 7.32 | 97.11 ± 8.40 |  |
| **fixed effects** | **d.f.** | **F** | **p-value** |
| habitat | 1 | 6.551 | **0.011** |
| year | 12 | 26.641 | **<0.001** |
| habitat × year | 12 | 4.337 | **<0.001** |

**Table S3**. Correlation between First Clutch Laying Date (FCLD) and the urbanisation index in the city. *Significant effects (in bold) were tested using Anova type III performed on the results of a linear mixed model (LMM). The model included FCLD (expressed as Julian day, with 1 = January 1st) as response variable, and urbanisation index, year (discrete predictor with 13 levels) and their interaction as explanatory variables. Female and nest-box identity were added as random effects. The urbanisation index was defined as the proportion of impervious surface area (ISA) within a 100-m diameter around each nest-box, ranging from 0 (no impervious surface) to 1 (fully impervious surface).*

|  | **FCLD (n = 1184)** | |  |
| --- | --- | --- | --- |
| **fixed effects** | **d.f.** | **F** | **p-value** |
| urbanisation index | 1 | 4.577 | **0.033** |
| year | 12 | 14.584 | **<0.001** |
| urbanisation index × year | 12 | 2.119 | **0.014** |


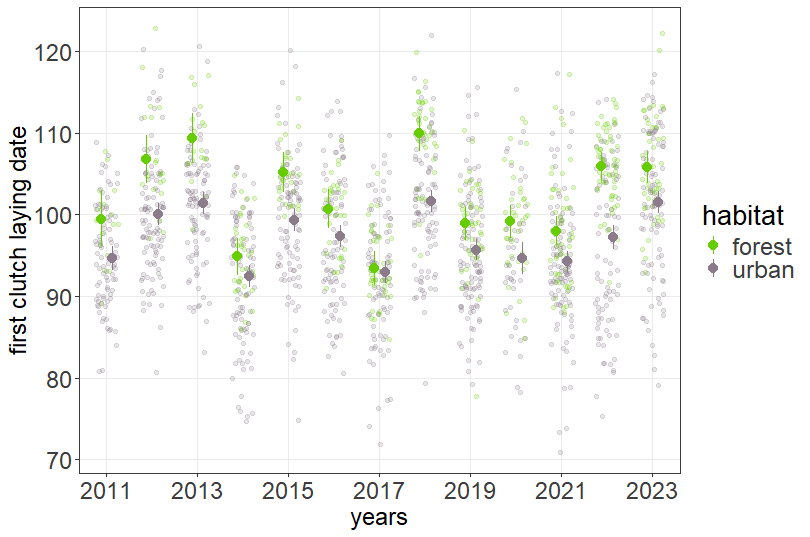


**Figure S2**. Average annual First Clutch Laying Date (FCLD) in the forest (in green) and in the city (in grey) across the monitoring years (2011 to 2023). *Values are outputs from a linear mixed model (LMM) which included an interaction between habitat and year, with FCLD (expressed as Julian day, with 1 = January 1st) as the response variable. Fixed effects included habitat (forest or urban), year (discrete predictor with 13 levels), and their interaction, while female and nest-box identities were included as random effects.*

**Table S4**. Linear selection gradients for First Clutch Laying Date (FCLD) based on an estimate of reproductive output that included a) first clutches only and b) first and second clutches together. *Linear selection gradients were estimated using models fitted separately for each habitat (left-hand side of table), while the differences in selection for the FCLD between habitats were tested using a model including both habitats (right-hand side of table). Significant (p < 0.05) and trend (0.05 < p < 0.10) effects are respectively in bold and italics and were tested using linear mixed models. Selection gradients quantify the strength and direction of selection acting on FCLD, while accounting for indirect selection on correlated traits; namely, Clutch Size (CS), which was included in our models. In analysis b), for females that laid two clutches in the same season, clutch size and reproductive output were respectively calculated as the total number of eggs and fledglings across both attempts. Note that FCLD always referred to the first egg laid during the first attempt.*

|  |  |  |  | **linear selection gradients** | | | | |  | **difference between habitats** | | | | |
| --- | --- | --- | --- | --- | --- | --- | --- | --- | --- | --- | --- | --- | --- | --- |
| **fitness estimation** | **habitat** | **sample size** | **trait** | **estimate** | **std. error** | **d.f.** | **t value** | **p-value** |  | **estimate** | **std. error** | **d.f.** | **t value** | **p-value** |
| a) first clutches | forest | 448 | FCLD  CS  FCLD  CS | -0.046 | 0.038 | 306.609 | -1.199 | 0.231 |  | *-0.068* | *0.040* | *1379.000* | *-1.691* | *0.091* |
|  |  |  |  | **0.112** | **0.031** | **439.749** | **3.652** | **<0.001** |  | *-* | *-* | *-* | *-* | *-* |
|  | urban | 1009 |  | -0.001 | 0.025 | 930.325 | -0.056 | 0.955 |  | - | - | - | - | - |
|  |  |  |  | **0.179** | **0.024** | **977.113** | **7.548** | **<0.001** |  | - | - | - | - | - |
|  |  |  |  |  |  |  |  |  |  |  |  |  |  |  |
| b) all clutches | forest | 448 | FCLD  CS  FCLD  CS | -0.048 | 0.034 | 305.884 | -1.436 | 0.152 |  | *-0.066* | *0.037* | *1404.693* | *1.778* | *0.076* |
|  |  |  |  | **0.400** | **0.027** | **377.818** | **15.090** | **<0.001** |  | *-* | *-* | *-* | *-* | *-* |
|  | urban | 1009 |  | -0.007 | 0.024 | 916.958 | -0.300 | 0.764 |  | - | - | - | - | - |
|  |  |  |  | **0.332** | **0.022** | **884.017** | **15.260** | **<0.001** |  | - | - | - | - | - |

**Table S5**. Quadratic selection differentials for First Clutch Laying Date (FCLD) based on an estimate of reproductive output that included a) first clutches only and b) first and second clutches together. *Quadratic selection differentials were estimated using models fitted separately for each habitat (left-hand side of table), while the differences in selection for the FCLD between habitats were tested using a model including both habitats (right-hand side of table). Significant (p < 0.05) and trend (0.05 < p < 0.10) effects are respectively in bold and italics and were tested using linear mixed models*.

|  |  |  | **quadratic selection differentials** | | | | |  | **difference between habitats** | | | | |
| --- | --- | --- | --- | --- | --- | --- | --- | --- | --- | --- | --- | --- | --- |
| **fitness estimation** | **habitat** | **sample size** | **estimate** | **std. error** | **d.f.** | **t value** | **p-value** |  | **estimate** | **std. error** | **d.f.** | **t value** | **p-value** |
| a) first clutches | forest | 455 | -0.009 | 0.024 | 420.076 | -0.361 | 0.719 |  | 0.010 | 0.031 | 1408.000 | 0.278 | 0.759 |
|  | urban | 1016 | 0.008 | 0.017 | 981.677 | 0.477 | 0.633 |  | - | - | - | - | - |
|  |  |  |  |  |  |  |  |  |  |  |  |  |  |
| b) all clutches | forest | 455 | 0.016 | 0.025 | 445.003 | 0.619 | 0.536 |  | -0.025 | 0.032 | 1427.606 | -0.784 | 0.432 |
|  | urban | 1016 | 0.015 | 0.018 | 993.791 | 0.847 | 0.397 |  | - | - | - | - | - |

**Table S6**. Quadratic selection gradients for First Clutch Laying Date (FCLD) based on an estimate of reproductive output that included a) first clutches only and b) first and second clutches together. *Quadratic selection gradients were estimated using models fitted separately for each habitat (left-hand side of table), while the differences in selection for the FCLD between habitats were tested using a model including both habitats (right-hand side of table). Significant (p < 0.05) and trend (0.05 < p < 0.10) effects are shown in bold and italics respectively and were tested using linear mixed models. Selection gradients quantify the strength and direction of selection acting on FCLD, while accounting for indirect selection on correlated traits; namely, Clutch Size (CS), which was included in our models. In analysis b), for females that laid two clutches in the same season clutch size and reproductive output were respectively calculated as the total number of eggs and fledglings across both attempts.*

|  |  |  |  | **quadratic selection gradients** | | | | |  | **difference between habitats** | | | | |
| --- | --- | --- | --- | --- | --- | --- | --- | --- | --- | --- | --- | --- | --- | --- |
| **fitness estimation** | **habitat** | **sample size** | **trait** | **estimate** | **std. error** | **d.f.** | **t value** | **p-value** |  | **estimate** | **std. error** | **d.f.** | **t value** | **p-value** |
| a) first clutches | forest | 448 | FCLD | 0.002 | 0.024 | 417.458 | 0.096 | 0.924 |  | 0.021 | 0.030 | 1396 | 0.674 | 0.501 |
|  |  |  | CS | *-0.026* | *0.015* | *361.118* | *-1.699* | *0.090* |  | *-* | *-* | *-* | *-* | *-* |
|  | urban | 1009 | FCLD | 0.009 | 0.017 | 976.216 | 0.503 | 0.616 |  | - | - | - | - | - |
|  |  |  | CS | **-0.029** | **0.014** | **989.450** | **-2.049** | **0.041** |  | - | - | - | - | - |
|  |  |  |  |  |  |  |  |  |  |  |  |  |  |  |
| b) all clutches | forest | 448 | FCLD | 0.003 | 0.022 | 418.418 | 0.131 | 0.896 |  | -0.022 | 0.028 | 1410.000 | -0.780 | 0.436 |
|  |  |  | CS | 0.010 | 0.022 | 362.966 | 0.475 | 0.635 |  | *-* | *-* | *-* | *-* | *-* |
|  | urban | 1009 | FCLD | 0.00008 | 0.016 | 981.000 | 0.005 | 0.996 |  | - | - | - | - | - |
|  |  |  | CS | 0.003 | 0.013 | 850.600 | -0.213 | 0.832 |  | - | - | - | - | - |

**Table S7.** Determinants of the probability of multiple brooding in urban and forest great tits. *Significant (in bold) effects were tested using Anova type III. Model included the probability of initiating a second clutch as a binomial response variable (1 = initiated a second clutch, 0 = did not), and* First Clutch Laying Date (FCLD)*, habitat (forest* vs. *urban) and their interaction as explanatory variables. Female identity, nest-box number and year were added as random effects.*

| **fixed effects** | **estimate ± std. error** | **d.f.** | **chisq** | **p-value** |
| --- | --- | --- | --- | --- |
| FCLD | -0.29 ± 0.14 | 1 | 4.34 | **0.037** |
| habitat | -1.43 ± 0.20 | 1 | 51.63 | **< 0.001** |
| FCLD × habitat | -0.64 ± 0.18 | 1 | 12.34 | **< 0.001** |

**Table S8**. Linear mixed models estimating year-specific **selection differentials and selection gradients** on standardised First Clutch Laying Date (FCLD) based on annual fledgling production from first broods only in forest population. *Selection differentials quantify the total association between FCLD and fitness, whereas selection gradients control for correlated reproductive traits, here Clutch Size (CS). Selection differentials were estimated from models including fixed effects of first clutch laying date (FCLD), breeding year, and their interaction (FCLD × year), allowing selection on laying date to vary among years. Selection gradients were estimated from analogous models additionally controlling for clutch size (CS). Female identity and nest-box number were included as random intercepts. Estimates, standard errors, degrees of freedom,* t *values and associated* p *values are reported. Significant effects are represented in bold and trend effects in italic.*

|  | **linear selection differentials** | | | | |  | **linear selection gradients** | | | | |
| --- | --- | --- | --- | --- | --- | --- | --- | --- | --- | --- | --- |
|  | **estimate** | **std. error** | **d.f.** | **t value** | **p-value** |  | **estimate** | **std. error** | **d.f.** | **t value** | **p-value** |
| intercept | **1.568** | **0.190** | **376.877** | **8.247** | **<0.001** |  | **1.535** | **0.187** | **368.941** | **8.223** | **<0.001** |
| FCLD | 0.378 | 0.242 | 376.886 | 1.565 | 0.118 |  | 0.369 | 0.237 | 369.010 | 1.558 | 0.120 |
| CS | **-** | **-** | **-** | **-** | **-** |  | **0.109** | **0.031** | **406.691** | **3.545** | **<0.001** |
| year |  |  |  |  |  |  |  |  |  |  |  |
| 2012 | **-0.768** | **0.262** | **364.041** | **-2.935** | **0.004** |  | **-0.738** | **0.257** | **357.702** | **-2.867** | **0.004** |
| 2013 | **-0.708** | **0.324** | **412.067** | **-2.186** | **0.029** |  | **-0.750** | **0.324** | **402.437** | **-2.318** | **0.021** |
| 2014 | **-0.984** | **0.267** | **404.828** | **-3.688** | **<0.001** |  | **-0.934** | **0.266** | **401.462** | **-3.512** | **<0.001** |
| 2015 | **-0.710** | **0.240** | **417.549** | **-2.952** | **0.003** |  | **-0.586** | **0.239** | **409.465** | **-2.451** | **0.015** |
| 2016 | **-0.965** | **0.223** | **414.107** | **-4.327** | **<0.001** |  | **-0.916** | **0.219** | **406.471** | **-4.180** | **<0.001** |
| 2017 | **-0.774** | **0.280** | **420.280** | **-2.767** | **0.006** |  | **-0.729** | **0.275** | **413.657** | **-2.653** | **0.008** |
| 2018 | -0.141 | 0.259 | 419.103 | -0.544 | 0.587 |  | -0.188 | 0.255 | 415.296 | -0.739 | 0.460 |
| 2019 | **-0.628** | **0.218** | **402.272** | **-2.883** | **0.004** |  | **-0.592** | **0.215** | **397.375** | **-2.757** | **0.006** |
| 2020 | **-0.562** | **0.216** | **408.139** | **-2.603** | **0.010** |  | **-0.526** | **0.212** | **400.467** | **-2.477** | **0.014** |
| 2021 | **-0.893** | **0.213** | **398.805** | **-4.184** | **<0.001** |  | **-0.837** | **0.210** | **392.110** | **-3.984** | **<0.001** |
| 2022 | *-0.369* | *0.223* | *402.714* | *-1.657* | *0.098* |  | -0.331 | 0.219 | 396.114 | -1.509 | 0.132 |
| 2023 | **-0.578** | **0.220** | **396.027** | **-2.631** | **0.009** |  | **-0.515** | **0.217** | **387.124** | **-2.378** | **0.018** |
| FCLD × year |  |  |  |  |  |  |  |  |  |  |  |
| 2012 | *-0.549* | *0.292* | *384.485* | *-1.882* | *0.061* |  | *-0.502* | *0.287* | *376.553* | *-1.753* | *0.080* |
| 2013 | **-0.661** | **0.329** | **390.347** | **-2.013** | **0.045** |  | *-0.579* | *0.340* | *388.835* | *-1.702* | *0.090* |
| 2014 | **-0.854** | **0.296** | **405.813** | **-2.891** | **0.004** |  | **-0.867** | **0.292** | **397.834** | **-2.967** | **0.003** |
| 2015 | -0.323 | 0.329 | 417.259 | -0.982 | 0.327 |  | -0.329 | 0.323 | 408.650 | -1.017 | 0.310 |
| 2016 | *-0.497* | *0.299* | *427.784* | *-1.664* | *0.097* |  | -0.483 | 0.293 | 419.013 | -1.649 | 0.100 |
| 2017 | -0.431 | 0.290 | 417.123 | -1.487 | 0.138 |  | -0.414 | 0.285 | 409.364 | -1.454 | 0.147 |
| 2018 | -0.398 | 0.278 | 422.779 | -1.433 | 0.153 |  | -0.350 | 0.273 | 414.927 | -1.281 | 0.201 |
| 2019 | -0.413 | 0.270 | 421.549 | -1.531 | 0.126 |  | -0.364 | 0.265 | 414.490 | -1.371 | 0.171 |
| 2020 | **-0.650** | **0.267** | **408.938** | **-2.437** | **0.015** |  | **-0.624** | **0.262** | **399.720** | **-2.386** | **0.018** |
| 2021 | -0.351 | 0.261 | 401.448 | -1.344 | 0.180 |  | -0.325 | 0.256 | 394.010 | -1.269 | 0.205 |
| 2022 | *-0.515* | *0.279* | *402.899* | *-1.844* | *0.066* |  | *-0.481* | *0.274* | *395.442* | *-1.753* | *0.080* |
| 2023 | -0.303 | 0.265 | 387.085 | -1.142 | 0.254 |  | -0.283 | 0.260 | 379.126 | -1.089 | 0.277 |

**Table S9.** Linear mixed models estimating year-specific **selection differentials and selection gradients** on standardised First Clutch Laying Date (FCLD) based on annual fledgling production from first broods only in urban population. *Selection differentials quantify the total association between FCLD and fitness, whereas selection gradients control for correlated reproductive traits, here Clutch Size (CS). Selection differentials were estimated from models including fixed effects of first clutch laying date (FCLD), breeding year, and their interaction (FCLD × year), allowing selection on laying date to vary among years. Selection gradients were estimated from analogous models additionally controlling for clutch size (CS). Female identity and nest-box number were included as random intercepts. Estimates, standard errors, degrees of freedom,* t *values and associated* p *values are reported. Significant effects are represented in bold and trend effects in italic.*

|  | **linear selection differentials** | | | | |  | **linear selection gradients** | | | | |
| --- | --- | --- | --- | --- | --- | --- | --- | --- | --- | --- | --- |
|  | **estimate** | **std. error** | **d.f.** | **t value** | **p-value** |  | **estimate** | **std. error** | **d.f.** | **t value** | **p-value** |
| intercept | **1.458** | **0.087** | **959.854** | **16.790** | **<0.001** |  | **1.401** | **0.085** | **952.342** | **16.558** | **<0.001** |
| FCLD | 0.047 | 0.111 | 925.591 | 0.422 | 0.673 |  | 0.070 | 0.108 | 922.472 | 0.649 | 0.516 |
| CS | **-** | **-** | **-** | **-** | **-** |  | **0.183** | **0.024** | **954.985** | **7.640** | **<0.001** |
| year |  |  |  |  |  |  |  |  |  |  |  |
| 2012 | *-0.212* | *0.120* | *858.908* | *-1.760* | *0.079* |  | -0.117 | 0.118 | 854.257 | -0.990 | 0.322 |
| 2013 | -0.191 | 0.139 | 924.516 | -1.376 | 0.169 |  | -0.177 | 0.135 | 919.324 | -1.314 | 0.189 |
| 2014 | **-0.671** | **0.133** | **898.337** | **-5.046** | **<0.001** |  | **-0.647** | **0.130** | **892.307** | **-4.990** | **<0.001** |
| 2015 | *-0.232* | *0.127* | *916.384* | *-1.821* | *0.069* |  | -0.106 | 0.126 | 912.195 | -0.840 | 0.401 |
| 2016 | **-0.534** | **0.119** | **894.041** | **-4.484** | **<0.001** |  | **-0.474** | **0.117** | **890.503** | **-4.058** | **<0.001** |
| 2017 | **-0.962** | **0.119** | **895.706** | **-8.111** | **<0.001** |  | **-0.904** | **0.116** | **890.646** | **-7.810** | **<0.001** |
| 2018 | **-0.590** | **0.118** | **911.113** | **-5.017** | **<0.001** |  | -0.547 | 0.115 | 905.950 | -4.774 | **<0.001** |
| 2019 | **-0.734** | **0.111** | **916.130** | **-6.609** | **<0.001** |  | **-0.665** | **0.109** | **911.749** | **-6.129** | **<0.001** |
| 2020 | **-0.285** | **0.138** | **895.406** | **-2.062** | **0.039** |  | -0.208 | 0.136 | 885.829 | -1.532 | 0.126 |
| 2021 | **-0.804** | **0.113** | **920.833** | **-7.116** | **<0.001** |  | **-0.717** | **0.111** | **917.160** | **-6.478** | **<0.001** |
| 2022 | **-0.327** | **0.127** | **944.635** | **-2.577** | **0.010** |  | **-0.252** | **0.124** | **938.325** | **-2.039** | **0.042** |
| 2023 | **-0.555** | **0.117** | **941.372** | **-4.732** | **<0.001** |  | **-0.407** | **0.116** | **938.612** | **-3.520** | **<0.001** |
| FCLD × year |  |  |  |  |  |  |  |  |  |  |  |
| 2012 | 0.000 | 0.144 | 898.558 | -0.001 | 0.999 |  | -0.016 | 0.140 | 896.167 | -0.116 | 0.908 |
| 2013 | 0.140 | 0.162 | 924.223 | 0.864 | 0.388 |  | 0.135 | 0.158 | 921.563 | 0.856 | 0.392 |
| 2014 | -0.132 | 0.148 | 921.701 | -0.892 | 0.372 |  | -0.185 | 0.144 | 918.489 | -1.280 | 0.201 |
| 2015 | -0.027 | 0.148 | 939.794 | -0.183 | 0.854 |  | -0.092 | 0.145 | 938.888 | -0.636 | 0.525 |
| 2016 | 0.097 | 0.142 | 930.286 | 0.686 | 0.493 |  | 0.100 | 0.140 | 927.807 | 0.717 | 0.473 |
| 2017 | -0.198 | 0.137 | 928.445 | -1.437 | 0.151 |  | -0.178 | 0.134 | 925.358 | -1.329 | 0.184 |
| 2018 | -0.119 | 0.136 | 919.613 | -0.876 | 0.381 |  | -0.137 | 0.133 | 915.623 | -1.035 | 0.301 |
| 2019 | -0.143 | 0.138 | 941.577 | -1.040 | 0.298 |  | -0.141 | 0.134 | 936.989 | -1.049 | 0.294 |
| 2020 | -0.091 | 0.155 | 959.876 | -0.590 | 0.555 |  | -0.084 | 0.150 | 954.855 | -0.562 | 0.574 |
| 2021 | -0.071 | 0.133 | 930.193 | -0.535 | 0.593 |  | -0.072 | 0.130 | 926.561 | -0.554 | 0.579 |
| 2022 | -0.037 | 0.134 | 958.975 | -0.276 | 0.782 |  | -0.033 | 0.130 | 955.465 | -0.252 | 0.801 |
| 2023 | -0.081 | 0.132 | 944.737 | -0.615 | 0.538 |  | -0.128 | 0.128 | 940.878 | -1.001 | 0.317 |

**Table S10**. Standardized path coefficients from structural equation models (SEMs) conducted separately in forest and urban habitats. *Each row shows the effect of a predictor variable (e.g. First Clutch Laying Date; FCLD) on a response variable (e.g. Annual Egg Number or annual number of fledglings), based on linear or generalized linear mixed models. The column* estimate *indicates the unstandardized model coefficient (logit scale for binomial models), while* std.estimate *provides the corresponding standardized coefficient used for effect size comparison across variables. Standard errors (*std.error*), degrees of freedom (*df*), test statistics (*crit.value*; t- or z-values), and associated p-values (*p.value*) are also reported. Significant effects (p < 0.05) are in bold. These models allow decomposition of direct and indirect pathways linking first clutch laying date (FCLD), multiple brooding, Annual Egg Number (AEN), and annual fledgling number (see Figure 4* *for a visual representation of these relationships).*

| **habitat** | **response** | **predictor** | **estimate** | **std.error** | **df** | **crit.value** | **p-value** | **std.estimate** |
| --- | --- | --- | --- | --- | --- | --- | --- | --- |
| forest | multiple brooding | FCLD | **-0.341** | **0.160** | **455.000** | **-2.137** | **0.0326** | **-0.180** |
|  | AEN | multiple_brooding | **2.074** | **0.055** | **318.516** | **37.513** | **<0.001** | **0.843** |
|  | AEN | FCLD | **-0.058** | **0.028** | **145.620** | **-2.048** | **0.0424** | **-0.058** |
|  | number of fledglings | FCLD | -0.048 | 0.034 | 305.885 | -1.436 | 0.1521 | -0.068 |
|  | number of fledglings | AEN | **0.400** | **0.027** | **377.818** | **15.090** | **<0.001** | **0.564** |
|  |  |  |  |  |  |  |  |  |
| city | multiple brooding | FCLD | **-0.861** | **0.153** | **1028.000** | **-5.643** | **<0.001** | **-0.395** |
|  | AEN | multiple_brooding | **2.680** | **0.073** | **746.550** | **36.936** | **<0.001** | **0.731** |
|  | AEN | FCLD | **-0.069** | **0.023** | **851.866** | **-3.032** | **0.0025** | **-0.069** |
|  | number of fledglings | FCLD | -0.007 | 0.024 | 916.958 | -0.300 | 0.7642 | -0.009 |
|  | number of fledglings | AEN | **0.332** | **0.022** | **884.017** | **15.256** | **<0.001** | **0.425** |

**Table S11.** Linear selection gradients for First Clutch Laying Date (FCLD), Clutch Size (CS), and Multiple Brooding (MB) based on reproductive output including all clutches. *Linear selection gradients were estimated using models fitted separately for each habitat (left-hand side of table), while differences in selection between habitats were tested using models including both habitats (right-hand side of table). Significant effects (p < 0.05) are shown in* ***bold****, and marginally significant trends (0.05 < p < 0.10) are* italicized*. Estimates were derived from linear mixed models. The traits included in the analysis were FCLD; Clutch Size (CS); and Multiple Brooding (MB), indicating whether a female laid more than one clutch during the same breeding season (1 = multiple brooding, 0 = single clutch only). Selection gradients quantify the strength and direction of selection acting on each trait while accounting for potential correlations between them. The MB variable was included as a covariate to disentangle selection acting on reproductive timing and clutch size from that associated with producing multiple broods. Reproductive output was measured as the total number of fledglings produced per female, across all clutches in the season.*

|  |  |  |  | **linear selection gradients** | | | | |  | **difference between habitats** | | | | |
| --- | --- | --- | --- | --- | --- | --- | --- | --- | --- | --- | --- | --- | --- | --- |
| **fitness estimation** | **habitat** | **sample size** | **trait** | **estimate** | **std. error** | **d.f.** | **t value** | **p-value** |  | **estimate** | **std. error** | **d.f.** | **t value** | **p-value** |
| all clutches | forest | 448 | FCLD | -0.054 | 0.033 | 314.080 | -1.652 | 0.100 |  | *0.064* | *0.037* | *1407.189* | *1.724* | *0.085* |
|  |  |  | CS | **0.202** | **0.051** | **421.024** | **3.954** | **<0.001** |  | *-* | *-* | *-* | *-* | *-* |
|  |  |  | MB | **0.553** | **0.121** | **420.397** | **4.555** | **<0.001** |  | *-0.136* | *0.106* | *1202.695* | *-1.282* | *0.200* |
|  | urban | 1009 | FCLD | -0.004 | 0.024 | 928.309 | -0.151 | 0.880 |  | - | - | - | - | - |
|  |  |  | CS | **0.259** | **0.033** | **981.793** | **7.846** | **<0.001** |  | - | - | - | - | - |
|  |  |  | MB | **0.357** | **0.118** | **965.660** | **3.024** | **0.003** |  | - | - | - | - | - |
